# Quantifying the compressibility of the human brain

**DOI:** 10.1073/pnas.2531115123

**Published:** 2026-01-21

**Authors:** Nicholas J. Weaver, Joshua Faskowitz, Richard F. Betzel, Christopher W. Lynn

**Affiliations:** ^a^Department of Physics, Yale University, New Haven, CT 06520; ^b^Quantitative Biology Institute, Yale University, New Haven, CT 06520; ^c^Program in Physical and Engineering Biology, Yale University, New Haven, CT 06520; ^d^Department of Psychological and Brain Sciences, Indiana University, Bloomington, IN 47405; ^e^Department of Neuroscience, University of Minnesota, Minneapolis, MN 55455; ^f^Masonic Institute for the Developing Brain, University of Minnesota, Minneapolis, MN 55414; ^g^Wu Tsai Institute, Yale University, New Haven, CT 06520

**Keywords:** minimax entropy, information theory, network neuroscience, statistical physics, fMRI

## Abstract

In the human brain, the allowed patterns of activity are constrained by the correlations between brain regions. Yet it remains unclear which correlations—and how many—are needed to predict large-scale neural activity. Here, we present an information-theoretic framework to identify the most important correlations, which provide the most accurate predictions of neural states. Applying our framework to cortical activity in humans, we find that the vast majority of variance in activity is explained by a small number of correlations. This means that the brain is highly compressible: Only a sparse network of correlations is needed to predict large-scale activity. We find that this compressibility is strikingly consistent across different individuals and cognitive tasks and that, counterintuitively, the most important correlations are not necessarily the strongest. Together, these results suggest that nearly all correlations are not needed to predict neural activity, and we provide the tools to uncover the key correlations that are.

## Significance

The correlations between different regions of the human brain define the shape of neural activity. While these correlations form the foundation for our understanding of human brain function, basic questions remain. How many correlations do we need to predict brain-wide activity? And which correlations are most important? To answer these questions, we develop a method to quantify the compressibility of the human brain. Across many humans and cognitive tasks, we find that the brain is highly compressible: Only a small number of important correlations are needed to capture neural activity. This reveals that the human brain may be simpler than previously thought, with potential implications for human cognition and disease.

Noninvasive neuroimaging has revolutionized our ability to record large-scale activity in the human brain (1). Individual regions, each reflecting the coarse-grained activity of a large population of neurons, interact to produce brain-wide activity that is responsible for learning, memory, perception, planning, and information processing (2–6). The functional networks formed by these regions (nodes) and the correlations between them (edges) have provided key insights into human cognition (7–10). Variations in these networks are linked to cognitive disorders, human development, and environmental factors (11–16). However, despite their crucial role in understanding cognition and disease, there remain basic questions about what correlations reveal about the nature of neural activity itself. Among all pairs of brain regions, which correlations are most important for predicting large-scale activity? And how many correlations do we need to construct an accurate model of the human brain?

Answering these questions is a problem of compression. The human brain is strongly correlated, with activity only exploring a small subset of all possible states (17–19). This suggests that a dense network of correlations is needed to constrain the neural activity. Alternatively, a small number of correlations may combine to have a large impact on the brain as a whole. In this simplified scenario, one would only need a sparse network of important correlations to predict the rest. If such a network exists, then the brain is highly compressible.

Information theory provides the foundation to make this intuition concrete (20, 21). Using the maximum entropy principle, one can map a network of correlations to predictions about neural activity (20, 22). For a given number of correlations, the optimal network (which yields the most accurate predictions) provides the best compression of the system (23). We show that this leads to a direct generalization of maximum entropy known as the minimax entropy principle (24–27), which we solve to identify the optimal networks of correlations. Applying our framework to cortex-wide activity in healthy human subjects, we find that only a small number of correlations are needed to predict large-scale patterns of activity. We therefore find that the brain is highly compressible.

## Constraining Activity with a Network of Correlations

Correlations tell us which regions are likely to activate together; once combined into a network they place complex constraints on the allowed patterns of activity. Consider N brain regions with collective activity defined by the vector $x=\{x_{i}\}$, where $x_{i}$ reflects the activity of region i. The activity of each region is z-scored so that the mean is zero and the variance is one; in this way, covariances and correlations are equivalent. Any distribution over the states P(x) defines a space of possible activity patterns. The size of this space—that is, our uncertainty about the neural states x—is quantified by the entropy S(P). In the simplest case, suppose we only measure the variance in activity $\sigma_{i}^{2}$ of each region in an experiment. The most unbiased distribution over states is composed of independent Gaussians (22, 28), and our uncertainty about the neural activity is defined by the independent entropy $S_{\text{ind}}=\frac{1}{2}\sum_{i}\log\sigma_{i}^{2}+\frac{N}{2}\;\text{log}\left(2\pi e\right)$. At the other extreme, in addition to the individual variances, consider the full set of covariances between regions $\Sigma_{\mathrm{ij}}$. By constraining the activity, these correlations reduce our uncertainty to the entropy of a fully connected Gaussian $S_{\text{tot}}=\frac{1}{2}\log\left|\Sigma \right|+\frac{N}{2}\;\text{log}\left(2\pi e\right)$, where |Σ| is the determinant of the covariance matrix (22, 28).

Between these two extremes, a subset of the covariances can be represented as a network G, with each edge (ij) reflecting a covariance $\Sigma_{\mathrm{ij}}$ included in our constraints (7, 8). Given such a network, the most unbiased prediction for the distribution over states is the one with maximum entropy (22, 28),

[1] $$\begin{array}{c} P_{G}\left(x\right)=\sqrt{\frac{\left|J\right|}{{\left(2\pi \right)}^{N}}}\exp(-\frac{1}{2}x^{T}Jx), \end{array}$$

where J is the precision matrix. In practice, for each covariance in the network G, one must compute the precision $J_{\mathrm{ij}}$ such that the model covariance ${\left(J^{-1}\right)}_{\mathrm{ij}}$ matches $\Sigma_{\mathrm{ij}}$, while all other elements of the precision remain zero. This means that the network G represents both the set of constrained covariances as well as the set of nonzero entries in the precision matrix. Note that we make no assumptions about the underlying connections between brain regions; rather, Eq. **1** provides the unique map from covariances to predictions for neural activity. The model is equivalent to a Gaussian graphical model (GGM) (29–32) for which there exist efficient inference algorithms (*Materials and Methods*) (33, 34). Thus, for a network of covariances G, our uncertainty about the collective activity is defined by the entropy $S_{G}=-\frac{1}{2}\log\left|J\right|+\frac{N}{2}\;\text{log}\left(2\pi e\right)$.

**Fig. 1.**
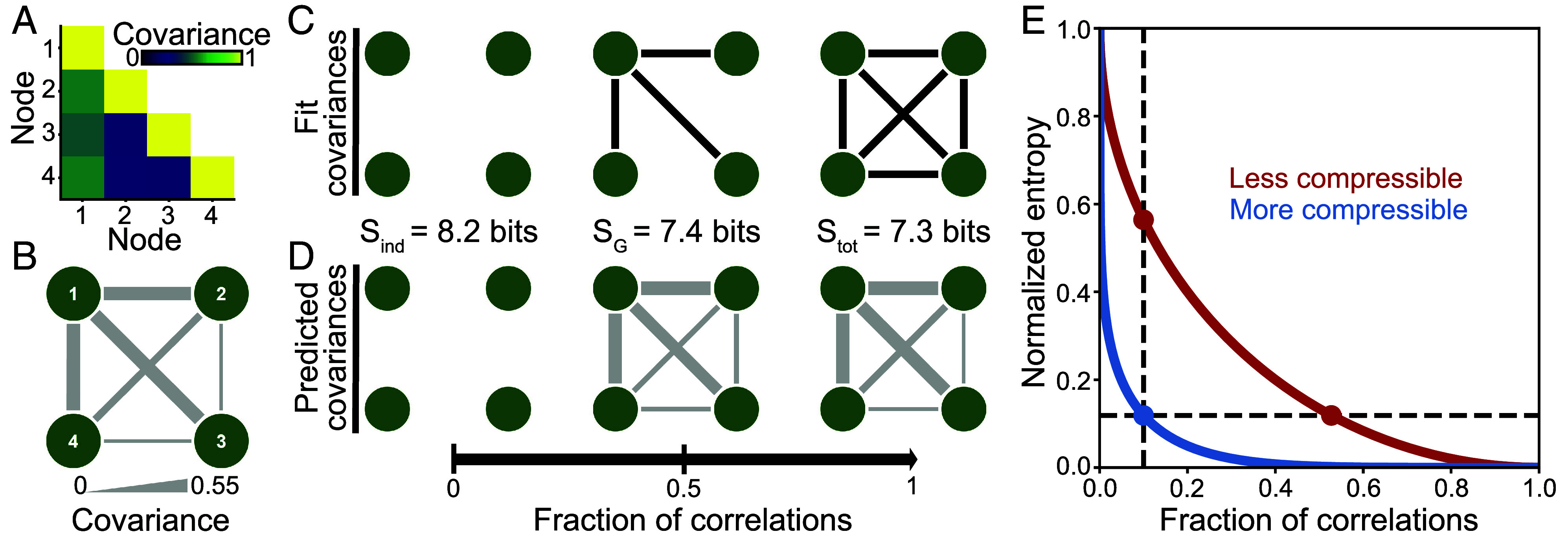
Quantifying uncertainty given a network of correlations. (*A* and *B*), Covariance matrix (*A*) and network representation (*B*) for a minimal system of four regions with z-scored activity (*Materials and Methods*). (*C* and *D*), Networks of correlations G (*C*) and the covariances predicted by the corresponding maximum entropy models $P_{G}$ (*D*). The network formed by the three strongest covariances produces accurate predictions for all covariances (*Middle*). (*E*) Illustration of the normalized entropy $\tilde{S}$ versus the fraction of correlations (or the density of the network G) for neural activity that is either more compressible (blue) or less compressible (red). In a more compressible system, one can achieve lower uncertainty for a given number of correlations (vertical line), and one can find a sparser network (with fewer correlations) to achieve a given level of uncertainty (horizontal line).

Consider a minimal example of four brain regions (Fig. 1 *A* and *B*). With no covariances, the regions behave independently with high uncertainty $S_{\text{ind}}$ (Fig. 1*C*, *Left*). With all of the covariances, we necessarily capture all of the correlation structure, reducing our uncertainty to $S_{\text{tot}}$ (Fig. 1*C*, *Right*). Between these limits, one might hope to find a small subset of covariances that produces a large reduction in uncertainty; that is, a good compression. Indeed, by constraining the three strongest covariances, the maximum entropy model accurately predicts the remaining indirect correlations (Fig. 1*D*), and the entropy $S_{G}$ nearly reduces to that of the fully connected network $S_{\text{tot}}$.

We therefore arrive at the following picture: As one includes more correlations in a network, the uncertainty about neural activity decreases, resulting in a hierarchy of entropy $S_{\text{ind}}\geq S_{G}\geq S_{\text{tot}}$. To compare across different datasets, we define the normalized entropy,

[2] $$\begin{array}{c} {\tilde{S}}_{G}=\frac{S_{G}-S_{\text{tot}}}{S_{\text{ind}}-S_{\text{tot}}}, \end{array}$$

which quantifies the progress from no correlations (${\tilde{S}}_{G}=1$) to a network that explains all of the structure in the neural activity (${\tilde{S}}_{G}=0$). If the brain is easier to compress, then for a given number of correlations, one should be able to find a network that reduces our uncertainty ${\tilde{S}}_{G}$ (Fig. 1*E*, *vertical line*). Equivalently, to achieve a given level of uncertainty, one should be able to find a network with fewer correlations (Fig. 1*E*, *horizontal line*). Thus, in order to quantify the compressibility of the human brain, we must first identify the optimal networks of correlations.

## Optimally Compressing Neural Activity

For a given number of correlations, we seek the network G that minimizes the entropy $S_{G}$. This is an instance of the “minimax entropy” principle, which has recently been proposed to construct optimal models of complex systems, but has yet to be applied to human neural activity (23–27). Notably, since $P_{G}$ is a maximum entropy model (Eq. **1**), the KL divergence between the model and the data simplifies to a difference in entropy $D_{\text{KL}}(P_{\text{exp}}\left|\right|P_{G})=S_{G}-S_{\text{exp}}$, where $P_{\text{exp}}\left(x\right)$ and $S_{\text{exp}}$ are the experimental distribution over states and entropy, respectively (*Materials and Methods*). Thus, the optimal network G (which minimizes the entropy $S_{G}$) also minimizes the divergence from data, thereby providing the most accurate description of the neural activity.

Given a desired number of correlations, identifying the optimal network poses two distinct challenges. First, for a given network G, one must compute the model $P_{G}$ that matches the covariances $\Sigma_{\mathrm{ij}}$ in G; this can be accomplished using efficient GGM algorithms (*Materials and Methods*) (33, 34). Second, one must search over all networks G to find the one that provides the best compression, minimizing the entropy $S_{G}$. However, the number of possible networks explodes combinatorially, making brute force search impossible. Instead, as is common in discrete optimization, we decompose the search into a sequence of local optimization problems (23, 35). Beginning with the empty network, we iteratively add the correlation that produces the largest decrease in entropy $S_{G}$, thus maximally reducing our uncertainty. When these drops in entropy become small, we can even expand in the weak-correlation limit analytically to further increase efficiency (*Materials and Methods*). We repeat this greedy algorithm until all correlations have been added. In doing so, we identify not only a single (locally) optimal network, but the sequence of all optimal compressions across all numbers of correlations. The result is a compression curve $\tilde{S}\left(f\right)$, which defines the minimum uncertainty that can be achieved with a given fraction of the correlations f (Fig. 1*E*).

**Fig. 2.**
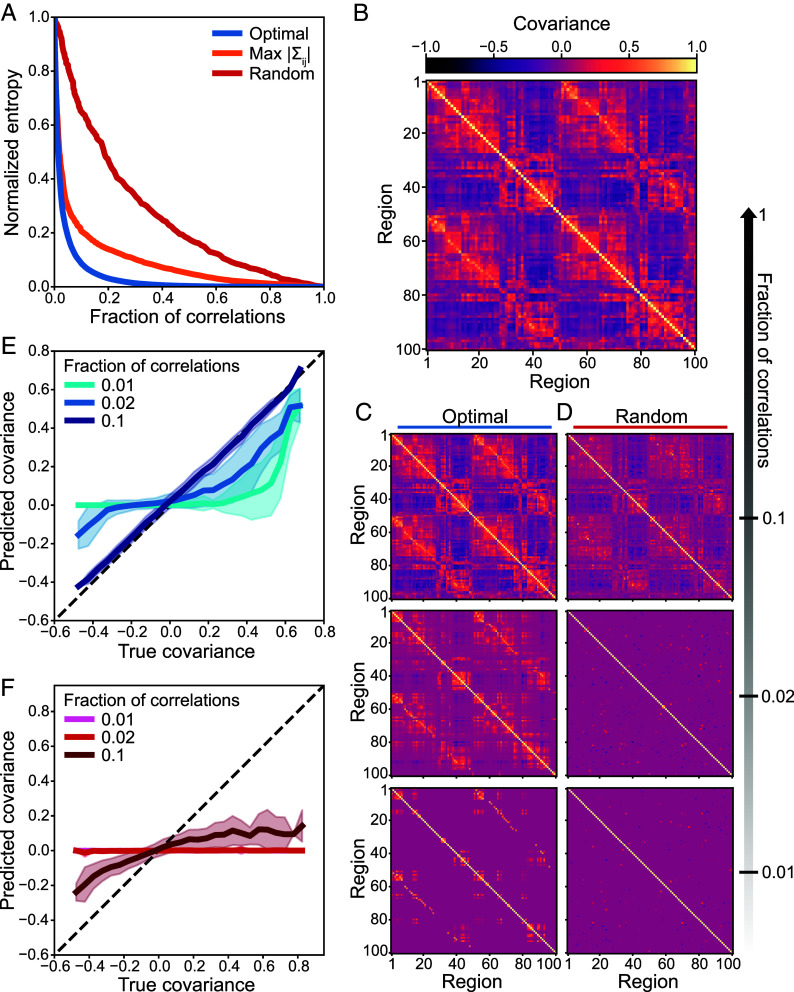
Human neural activity is highly compressible. (*A*) Normalized entropy ${\tilde{S}}_{G}$ as a function of the fraction of correlations in G for the optimal networks (blue), strongest correlations $|\Sigma_{\mathrm{ij}}|$ (orange), and random networks (red). (*B*) Covariances between brain regions measured in activity concatenated across all subjects and tasks. (*C* and *D*) Predicted covariances based on the optimal networks (*C*) and random networks (*D*) with different fractions of the correlations. (*E* and *F*) Predicted covariances versus their true values for models constructed from optimal networks (*E*) and random networks (*F*). Lines and shaded regions indicate means and SDs across all covariances that are not included in each model.

We apply our framework to cortex-wide activity from 99 healthy adults both at rest and across a suite of seven cognitive tasks, recorded using functional MRI (fMRI) as part of the Human Connectome Project (36). The collective activity consists of blood-oxygen-level-dependent (BOLD) fMRI signals from N=100 cortical parcels (37). Concatenating this activity across all subjects and tasks, we apply our minimax entropy algorithm to compute optimal compressions across all numbers of correlations. Strikingly, we find that with only a small number of correlations, the entropy drops significantly; only 1.4% of the correlations are needed to achieve a 50% reduction in entropy, and a 90% reduction only requires 9% of the correlations (Fig. 2*A*). Intuitively, one might expect the strongest correlations between regions to provide the tightest constraints on neural activity. To test this hypothesis, we compare against networks consisting of the strongest covariances. While these networks provide significantly better compressions than random correlations, the optimal networks still achieve up to orders of magnitude lower uncertainty (Fig. 2*A*). Together, these results indicate that the human brain is highly compressible, and that the optimal compressions themselves do not simply consist of the strongest correlations.

Given this compressibility, with only a small number of important correlations, we should be able to predict the entire correlation structure (Fig. 2*B*). For the optimal networks, as we increase the number of correlations, we see that the full structure quickly comes into focus (Fig. 2*C*). In fact, by fitting only 10% of the correlations between regions, the optimal model $P_{G}$ quantitatively predicts the remaining 90% (Fig. 2*E*). By contrast, with the same number of random correlations, most of the structure is lost (Fig. 2*D*), and the model significantly underestimates the true strengths of correlations (Fig. 2*F*). Thus, while the brain is strongly correlated (Fig. 2*B*), only a sparse network of these correlations is needed to explain the rest.

## Quantifying the Compressibility of Neural Activity

We are now prepared to quantify the compressibility of the brain. Rather than choosing a specific number of correlations, we would like compressibility to be a property of the neural activity itself. We therefore define compressibility to be the amount of uncertainty that can be removed via compression, averaged across all numbers of correlations,

[3] $$\begin{array}{c} C=1-\int_{0}^{1}\tilde{S}\left(f\right)df. \end{array}$$

**Fig. 3.**
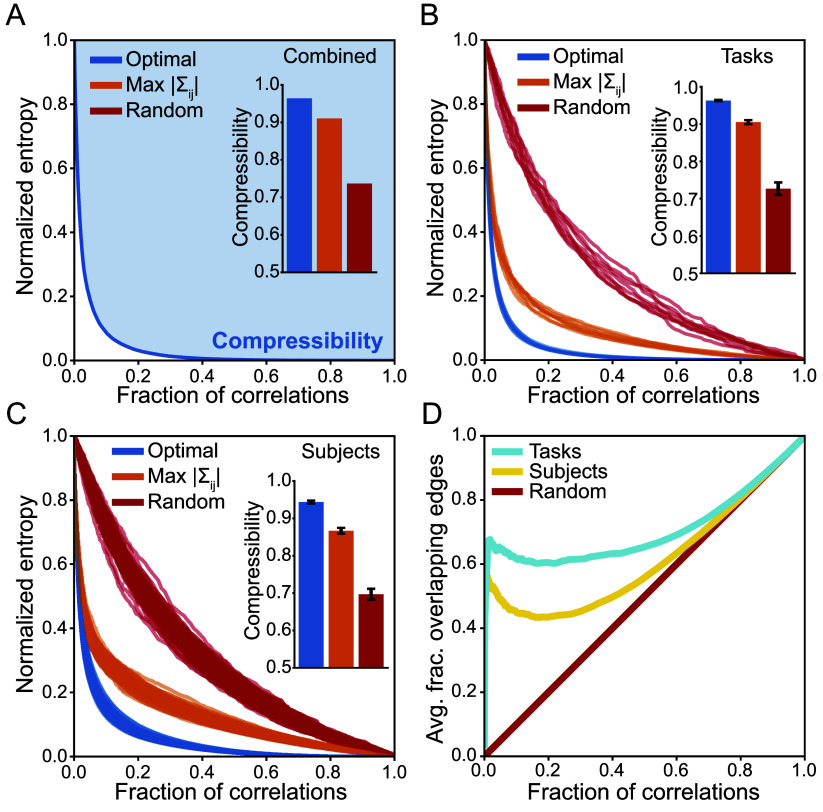
Quantifying compressibility across subjects and cognitive tasks. (*A*) Optimal normalized entropy $\tilde{S}\left(f\right)$ versus the fraction of correlations f for data combined across all tasks and subjects. Compressibility is the shaded area above the compression curve. The *Inset* displays compressibility values for optimal, maximum correlation, and random networks. (*A*–*C*) Normalized entropy versus fraction of correlations in optimal (blue), maximum correlation (orange), and random (red) networks for data within specific tasks (*B*) and within specific subjects (*C*). *Insets* display compressibility values averaged across tasks (*B*) and across subjects (*C*) with one-standard-deviation error bars. (*D*) Average overlap (fraction of shared correlations) between optimal networks for pairs of tasks (blue) and pairs of subjects (yellow) versus the fraction of correlations. The red line illustrates the overlap between random networks.

Visually, the compressibility represents the area above the compression curve (Fig. 3*A*). This approaches C=1 for perfectly compressible activity (with all structure concentrated on one correlation) and C=0 for perfectly incompressible activity (with all correlations needed to explain the structure). For the fMRI data combined across subjects and tasks, we find a compressibility C=0.96 near the theoretical maximum (Fig. 3*A*); meanwhile, suboptimal networks (composed of random or strong correlations) yield lower compressibilities. This means that, on average across all numbers of correlations, one can achieve a 96% reduction in uncertainty about the neural activity. Moreover, we confirm that this high compressibility is not simply due to the choice or number of brain regions; repeating the same analysis for a parcellation of N=200 regions leads to an even higher compressibility of C=0.98 (*SI Appendix*).

One might be concerned that concatenating activity across subjects and tasks introduces spurious correlations that lead to good compressions. Instead, we can investigate the compressibility of the brain within a given cognitive task (combined across subjects) or for a specific subject (combined across tasks, including rest). In both cases, our compression framework still uncovers sparse networks of correlations that produce steep drops in entropy (Fig. 3 *B* and *C*). Averaging across all numbers of correlations, we find that the brain is highly compressible both within tasks (C=0.96) and for individual subjects (C=0.94). Moreover, we see that the compression curves are surprisingly consistent across different tasks (Fig. 3*B*) and subjects (Fig. 3*C*), suggesting that the optimal networks themselves may be consistent. Indeed, for the top 10% of correlations, we find that the optimal networks for specific subjects overlap 53 times more strongly than random, and this overlap increases to 62 times more than random for different cognitive tasks (Fig. 3*D*). Given this consistency and given that they capture the vast majority of correlations, what is the network structure of optimal compressions?

## Structure of Optimal Correlations

**Fig. 4.**
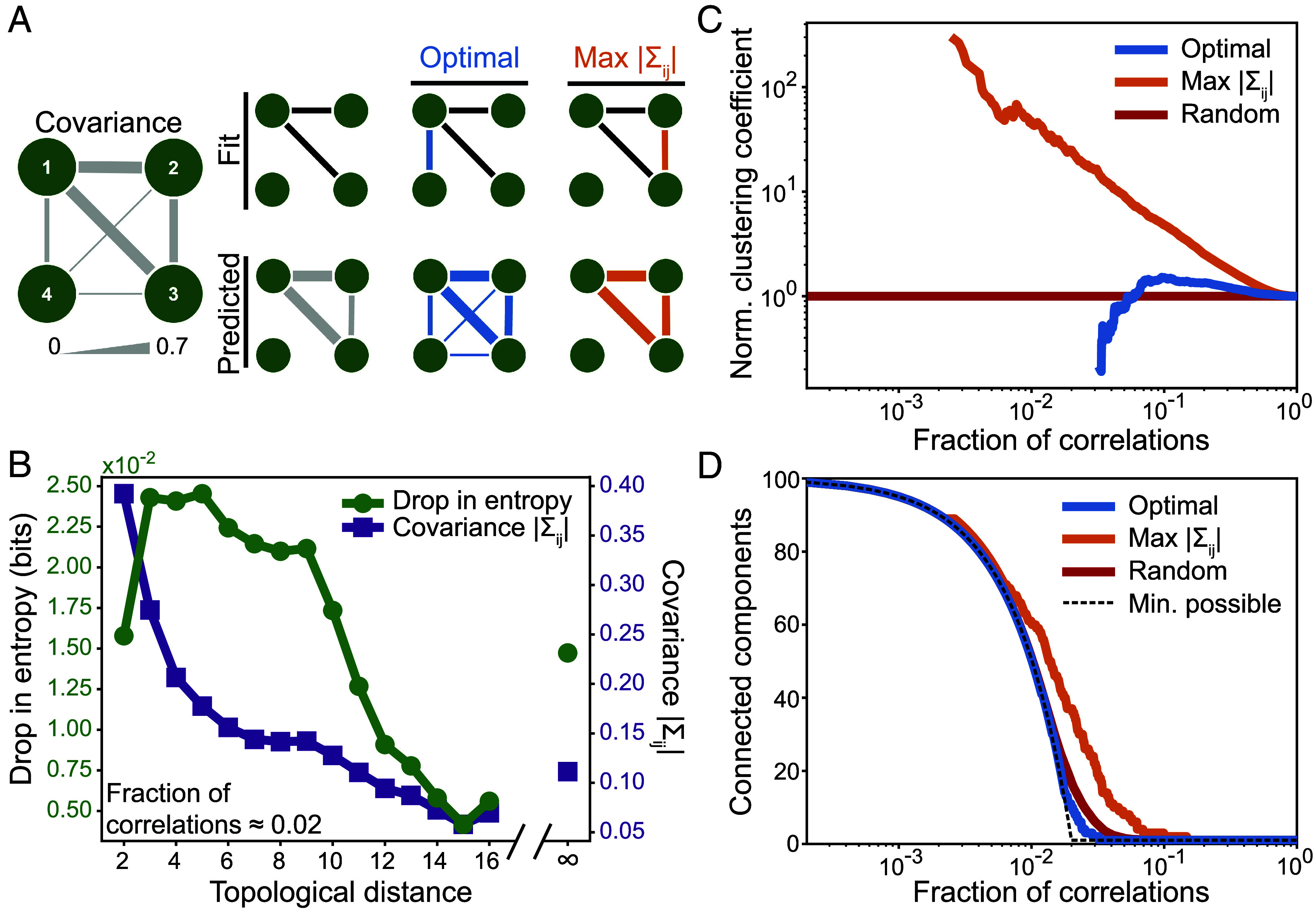
Network structure of optimal correlations. (*A*) For a minimal system of four regions, the first two optimal covariances are the strongest, yielding an accurate prediction for the third strongest (*Left*). Thus, the third strongest covariance is redundant, providing little improvement in accuracy (*Right*). The third optimal covariance is weaker, but provides more accurate predictions (*Middle*). (*B*) For the optimal model with 100 correlations, we plot the average drop in entropy from fitting covariances and average strength of covariances as a function of the topological distance between brain regions in the network (that is, the length of the shortest path). Topological distance is infinity if there does not exist a path between regions. (*C*) Global clustering coefficient (normalized by the density of the network) versus the fraction of correlations. Note that zero values are not shown. (*D*) Number of connected components versus the fraction of correlations, where each random value is averaged over 100 random networks. The dashed line indicates the minimum possible value. In (*A*–*C*), neural data are combined across all tasks and subjects.

We have seen that the optimal correlations between regions, which minimize our uncertainty about neural activity, are not simply the strongest (Figs. 2*A* andFig. 3 *B* and *C*). To understand how this is possible, consider the minimal system in Fig. 4*A*. With knowledge of the two strongest covariances $\Sigma_{12}$ and $\Sigma_{13}$, one can accurately predict the next strongest covariance $\Sigma_{23}$. Thus, when selecting the third correlation to include in the model, rather than choosing the strongest option $\Sigma_{23}$, one can gain more information by selecting a weaker covariance (in this case $\Sigma_{14}$). In general, while strong correlations tend to form tight loops (38), some of these may be predicted indirectly from the others; these strong but redundant correlations do not reduce our uncertainty about the neural activity.

This phenomenon plays a key role in shaping the network structure of optimal compressions. For example, for the optimal network with 100 correlations, we find that the strength of correlations decreases with increasing distance in the network (Fig. 4*B*). This means that selecting the strongest correlations leads to networks with many short loops, a property known as clustering (7). Meanwhile, the largest drops in entropy are not achieved by these strong, short-range correlations, but instead by correlations of intermediate strength and distance in the network (Fig. 4*B*). As a result, while the strongest correlations form networks with high clustering, the optimal networks exhibit orders of magnitude lower clustering (Fig. 4*C*). Rather than concentrating connectivity within tight clusters of regions, we find that optimal networks spread connectivity across distant parts of the network. This leads to the formation of a single giant component—wherein each region connects at least indirectly to every other region—with nearly as few correlations as possible (Fig. 4*D*) (39, 40).

**Fig. 5.**
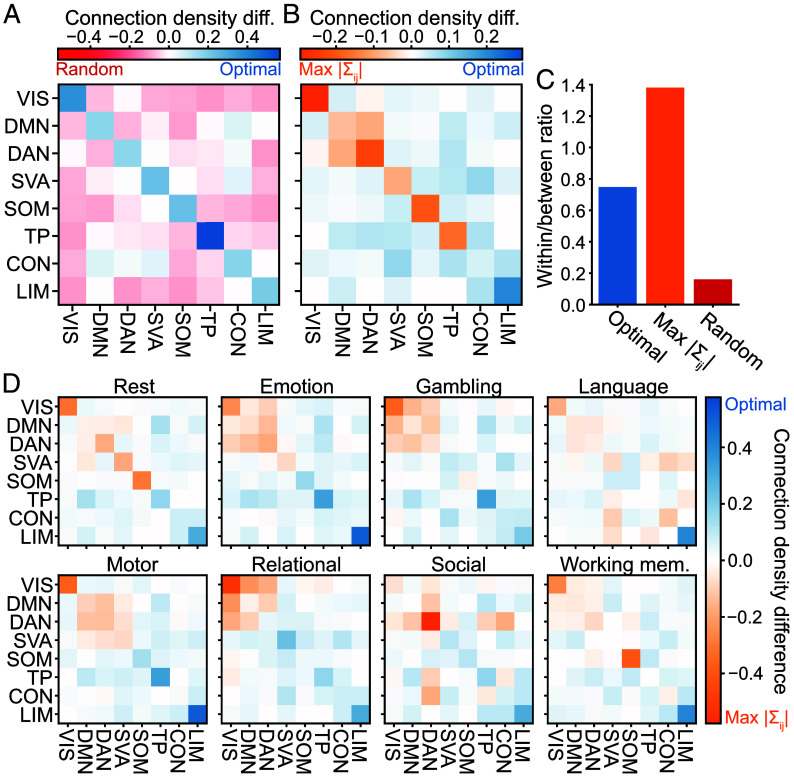
Systems-level structure of optimal compressions. (*A*), Difference in the density of connections between the optimal network (blue) and random networks (red) for pairs of regions in different cognitive systems. Diagonal (off-diagonal) elements indicate connections within (between) systems. (*B*), Difference in the density of connections between the optimal network (blue) and the network of strongest correlations (orange). (*C*), Ratio of correlations within versus between cognitive systems. In (*A*–*C*), neural data are combined across all tasks and subjects. (*D*), Difference in the density of connections between optimal and maximum correlation networks during specific cognitive tasks including rest (data combined across subjects). Across all panels, networks contain 10% of correlations. We coarse-grain regions into eight systems: visual (VIS), default mode (DMN), dorsal attention (DAN), salience/ventral attention (SVA), somatomotor (SOM), temporoparietal (TP), control (CON), and limbic (LIM).

Highly correlated brain regions are known to form functionally specific subnetworks known as cognitive systems (7, 8, 41). Our above results suggest that, rather than focusing on strong correlations within these systems, one might construct better models of neural activity by including weaker correlations between different cognitive systems. To test this hypothesis, we sort the 100 regions into eight subnetworks (37, 42). Correlations within each of these cognitive systems are much more likely to appear in optimal networks than random, indicating that they are important for constraining neural activity (Fig. 5*A*). However, relative to the strongest correlations, optimal networks tend to connect regions spanning different cognitive systems, suggesting that many of the strong correlations within systems are redundant (Fig. 5*B*). In fact, while the majority of the strongest correlations lie within specific systems, most of the optimal correlations span separate subnetworks (Fig. 5*C*).

We observe subtle deviations from this pattern in different cognitive tasks (Fig. 5*D*): Correlations between the visual (VIS), default mode (DMN), and dorsal attention (DAT) systems, which are highly engaged during visual and motor response tasks (43–45), provide less information about cortex-wide activity than one would expect from their strength alone. Meanwhile, correlations within the limbic (LIM) and temporoparietal (TEP) systems, which are associated with emotional and social processing (46, 47), are weak but important for constraining activity. Yet across all tasks, we consistently find that optimal compressions tend to spread connectivity between different cognitive systems (Fig. 5*D*). Together, these results demonstrate how, by avoiding tight clusters of strong but redundant correlations, one can achieve a much more effective compression of the human brain.

## Discussion

In the human brain, the correlations between brain regions have provided foundational insights into cognition and disease (1–16). But how many correlations—and which correlations—are needed to explain brain-wide activity remains poorly understood. To identify the most important correlations between brain regions, which generate the most accurate predictions of neural states, we show that one must solve the minimax entropy problem, which has roots in information theory and statistical physics (23–27). In fMRI data from a large cohort of subjects across several cognitive tasks (36), we find that only a small number of correlations are needed to explain the observed cortex-wide activity (Fig. 2). This reveals that the brain is highly compressible: One can ignore nearly all of the correlations between regions while still accurately predicting neural activity. In fact, we demonstrate that the compressibility of the human brain is near the theoretical maximum, and that this high compressibility is consistent across different subjects and cognitive tasks (Fig. 3). Finally, we find that the most important correlations for predicting neural activity are not simply the strongest, a fact that has profound implications for the structure of optimal compressions (Figs. 4 andFig. 5).

These results demonstrate that the constraints on neural activity in the human brain are distributed highly nonuniformly. A small fraction of the correlations between regions have orders of magnitude more influence on brain-wide activity than the vast majority of correlations. In turn, this suggests that the human brain may be dominated by an extremely sparse backbone of abnormally strong interactions. Indeed, such heavy-tailed distributions of connection strength are observed in the structural wiring between brain regions (48–50). At much smaller scales, similarly sparse networks have also been found to dominate both the correlations and physical connections between neurons (25–27, 51, 52). Identifying the interactions that dominate neural activity—which we have shown differ systematically from the strongest correlations—may have important implications for understanding neurological disorders and developing targeted treatments (7, 53–55).

Moreover, with the tools to quantify the compressibility of the human brain, a number of questions immediately arise. How does the brain’s high compressibility emerge during development (56, 57)? And how is it altered in diseases such as Alzheimer’s (58), Parkinson’s (59), and schizophrenia (60)? Moreover, how does the network and systems-level structure of optimal compressions vary across development and disease? With the explosion of available fMRI data, one can directly apply our compression framework to begin answering these questions. In fact, our methods can be used to study any continuous-valued recordings of neural activity, opening the door for future investigations of neural activity across different imaging modalities [including EEG and MEG (61, 62)] and even distinct species (63, 64). Across these settings, understanding how optimal compressions compare to other network inference techniques [such as dynamical causal modeling (65)] is a clear avenue for future research. Given the tendency of direct interactions to produce indirect correlations (Fig. 4*A*), we anticipate that many strong correlations in the brain may in fact be redundant, the natural consequences of neighboring interactions. In turn, we hypothesize that many neural systems will be amenable to simplified descriptions with only a small number of important correlations; that is, highly compressible.

## Materials and Methods

### Maximum Entropy.

Consider the matrix of covariances Σ between N brain regions. A subset of these covariances can be represented as a network G (Fig. 1). Given a network of correlations, the most unbiased prediction for the remaining covariances is provided by the maximum entropy model in Eq. **1**. To compute the maximum entropy model, for each edge (ij) in the network G, one must compute the entry of the precision matrix $J_{\mathrm{ij}}$ such that the corresponding model covariance ${\left(J^{-1}\right)}_{\mathrm{ij}}$ matches the experimental value $\Sigma_{\mathrm{ij}}$. There exist efficient algorithms for solving this problem, which are guaranteed to converge to the correct solution (33, 34, 66). Here, we use Algorithms 1 and 2 in ref. 33. Algorithm 1 iterates over entries of the precision matrix corresponding to edges in the network G, while Algorithm 2 iterates over elements of the model covariance corresponding to edges not in G. Thus, Algorithm 1 (Algorithm 2) is more efficient for networks with fewer (more) than roughly half the available edges. When calculating maximum entropy models, we switch from Algorithm 1 to 2 at 1,500 correlations, and we check that solutions converge within experimental errors (*SI Appendix*).

### Minimax Entropy.

Given a specified number of correlations, there are many different subsets—or networks—one could choose. Here, we show that the optimal network, which provides the most accurate model of the neural activity, also generates the best compression of the data. Letting $P_{\text{exp}}\left(x\right)$ denote the experimental distribution over states x, the Kullback–Leibler (KL) divergence (21) with a maximum entropy model $P_{G}\left(x\right)$ simplifies to a difference in entropy (23)

$$\begin{array}{ccc} D_{\text{KL}} & (P_{\text{exp}}\left|\right|P_{G})={\langle \log\frac{P_{\text{exp}}\left(x\right)}{P_{G}\left(x\right)}\rangle }_{\text{exp}} & \\ & ={\left\langle \log P_{\text{exp}}\left(x\right)\right\rangle }_{\text{exp}}-{\left\langle \log P_{G}\left(x\right)\right\rangle }_{\text{exp}} & \\ & =-S_{\text{exp}}+\frac{N}{2}\log\left(2\pi \right)-\frac{1}{2}\log\left(|J|\right)+\sum_{(ij)\in G}J_{\mathrm{ij}}{\left\langle x_{i}x_{j}\right\rangle }_{\text{exp}} & \\ & =-S_{\text{exp}}+\frac{N}{2}\log\left(2\pi \right)-\frac{1}{2}\log\left(|J|\right)+\sum_{(ij)\in G}J_{\mathrm{ij}}{\left\langle x_{i}x_{j}\right\rangle }_{P_{G}} & \\ & =-S_{\text{exp}}-{\left\langle \log P_{G}\left(x\right)\right\rangle }_{P_{G}} & \\ & =S_{G}-S_{\text{exp}}, \end{array}$$

where ${\left\langle \cdot \right\rangle }_{\text{exp}}$ represents an experimental average, and $S_{\text{exp}}$ is the entropy of $P_{\text{exp}}$. In the fourth equality, we are able to replace averages over $P_{\text{exp}}$ with averages over $P_{G}$ because the model is constrained to match the experimental correlations in G. We therefore find that minimizing the KL divergence is equivalent to minimizing the entropy $S_{G}$ of the maximum entropy model $P_{G}$. This minimax entropy principle tells us that the optimal network G, which provides the most accurate description of the activity, also produces the best compression of the data.

For a given number of correlations, the number of possible networks explodes combinatorially, making a brute force search for the optimal network impossible. Mathematically, this optimization is equivalent to constructing a Gaussian graphical model (GGM) with an $L_{0}$ regularization on the precision matrix, which is notoriously difficult. One heuristic for overcoming this challenge is using the graphical lasso, with an $L_{1}$ norm on the inverse covariance to penalize a lack of sparsity (67–70). Here, we instead decompose the problem into a sequence of local optimizations that we can solve exactly. Specifically, we develop a greedy algorithm for constructing the optimal network G. Starting from the independent model (with no correlations), we iteratively add the correlation to our model that produces the largest drop in entropy $S_{G}$. Using this greedy approach, we can identify the locally optimal network not only for a one number of correlations, but across all numbers of correlations, thus generating the entire entropy curve (Fig. 2). We confirm that this greedy algorithm outperforms many other heuristics (*SI Appendix*).

### Improving the Efficiency of Compression.

At each step of the greedy algorithm, we must fit $O(N^{2})$ different maximum entropy models: one for each of the possible correlations that could be added to the model. This process must then be repeated for each of the $O(N^{2})$ steps of the greedy algorithm. To scale our compression to large-scale data, we therefore need strategies for improving efficiency. To begin, we note that if the network has no loops, then each correlation produces a drop in entropy equal to the mutual information between the two brain regions (25, 26, 71). Thus, for sparse networks with no loops, one can simply select the correlations corresponding to the largest mutual information.

Once loops are included in the network, we require new strategies for efficiency. For this purpose, we derive an analytic approximation to the drop in entropy that does not require fitting a new maximum entropy model (*SI Appendix*). We use perturbation theory to estimate entropy drops in the limit of small errors on the predicted correlations (or, equivalently, in the limit of small precision entries). As the greedy algorithm progresses, and the network becomes denser, the entropy drops become very small (Fig. 2*A*), making our approximation accurate. In practice, we compute entropy drops exactly for the first 50 correlations before switching to our approximation. We confirm the accuracy of our approximation, even in sparse networks with only 2% of correlations (*SI Appendix*).

### Data.

We use fMRI data from the Human Connectome Project (HCP) from ref. 36 for 99 human subjects. The HCP study was approved by the Washington University Institutional Review Board and informed consent was obtained from all subjects. Participants were not compensated. The subjects were scanned at rest and while performing seven cognitive tasks: emotion, gambling, language, motor, relational, social, and working memory. When we refer to “tasks,” we include rest for convenience. A comprehensive description of the imaging parameters and image preprocessing can be found in ref. 72. Using a 3T Siemens Connectome Skyra with a 32-channel head coil, gradient-echo EPI images were acquired during eight conditions with the following parameters: TR = 720 ms; TE = 33.1 ms; 2-mm isotropic voxel resolution; flip angle = 52^°^; multiband acceleration factor = 8. See ref. 73 for details about the details and timing of each administered task. Minimally preprocessed data, which included slice timing, motion, and distortion correction, normalized to a common surface space template were downloaded. In addition, time series were linearly detrended, temporally filtered (0.008 to 0.08 Hz), and had 24 head motion parameters, eight mean signals from white matter and cerebrospinal fluid, and four global signals regressed out of the time series data, corresponding to strategy six in ref. 74. Denoised vertex-wise time series were averaged within each area of the Schaefer 100 parcellation (37) at each time step, to create parcel-wise time series.

We concatenate scans for each combination of subject and task and z-score them, subtracting the mean and dividing by the SD. Data were combined into larger timeseries in three ways: across all tasks and subjects (yielding combined data), across tasks for a given subject (yielding subject data), and across subjects for a given task (yielding task data). Note that all activity was mapped to the same atlas, making it meaningful to combine the time series from the same region in different subjects (37, 42). Scans for different tasks have different lengths, with the shortest (emotion) having just 176 frames. There were two scans per task per subject, and a maximum of four for rest. For task and subject data, two subjects were excluded due to having too few rest scans. These two subjects still had their data included in the combined data. For task data, to avoid differences due to sampling, we only use the beginning of each scan with the same length as that of the shortest task. For each task, two scans (including rest) were included per subject. Subject data included two scans per nonrest task and three rest scans per subject. This ensured the same amount of data for all subjects.

## Supplementary Material

Appendix 01 (PDF)

## Data Availability

Covariance matrices of preprocessed fMRI BOLD activity timeseries and python notebooks containing code to build minimax entropy models optimally, as well as randomly and using strongest correlations, have been deposited in GitHub (TBD, https://github.com/NicholasJWeaver/BrainCompressibility2025/) (75).
