# Supplementary material for: Quantifying the compressibility of the human brain
Source: Proc Natl Acad Sci U S A. 2026 Jan 21;123(4):e2531115123. doi: 10.1073/pnas.2531115123 (PMC12846795; doi:10.1073/pnas.2531115123)
Supplement: Supplementary file 1 — Appendix 01 (PDF) [file pnas.2531115123.sapp.pdf]

Supplemental Information

1 Speeding up the minimax entropy procedure, with additional details.

We need to find ways to speed up the greedy algorithm for building minimax entropy models. We can first use the result that if adding an edge (ij) does not create any loops in the graph, the entropy drop from adding that edge is simply the mutual information between the nodes in that edge: [1–3]

$$MI_{ij} = -\frac{1}{2}\log\left[1 - \frac{\Sigma_{ij}\Sigma_{ji}}{\Sigma_{ii}\Sigma_{jj}}\right] \quad (1)$$

This can be computed quickly and does not require us to find the maxent model itself.

As we grow the network of constrained correlations, the algorithm used to compute a particular maximum entropy model takes more and more time to run. We can speed up the algorithm by taking advantage of the fact that it iterates over one edge at a time, allowing us to rewrite the algorithm using results for low rank matrix updates. Even with this speedup, adding edges to the graph takes longer and longer. As we create more opportunities to add loops, we have fewer opportunities to use the mutual information trick and have to run the full maxent algorithm more and more.

To make progress, we developed a method for predicting the entropy drop that would result from adding any edge to the network without actually running the maxent algorithm (See section 2). Past a certain number of edges added, in this case 50, we switch from exactly finding the entropy drop from adding each candidate edge to predicting it. This allows us to then add the edge with the largest predicted entropy drop and run the maxent algorithm only once, to find the model corresponding to that new network of constraints. This model is then used in the process of predicting all of the entropy drops at the next step, and the process repeats. We can even increase the number of edges we add at a given time as the network of constraints becomes more dense. We add one edge per step until 200 edges added, then two per step until 400 edges, then five per step until 600 edges, then 10 per step until 800 edges, then 20 per step until 1000 edges, then 50 per step for the remainder of the process.

We use one final speedup. Maxent Algorithm 2,[4] which we discussed in the main text, runs quickly for sparse networks of edges but takes longer as the network becomes more dense. Algorithm 1[4] is slower for sparse networks but becomes faster for denser and denser networks. At some point for medium network density, in our case chosen to be 1500 edges added out of 4950, we switch from Algorithm 2 to Algorithm 1. With all of these steps, we are able to build minimax networks all the way from the independent model with no edges constrained to the full model with all of them constrained in a reasonable amount of time, on the order of hours.

2 Predicting the entropy drop from adding an edge.

We imagine wiggling one element of the covariance and seeing how the model entropy changes, while enforcing the constraints imposed by the entropy maximization procedure. We can perform a series expansion of the entropy change in terms of the error in the corresponding element of the covariance matrix. Here we denote the model covariance as K, with model precision $J = K^{-1}$, and the data covariance as Σ. We fit all diagonal elements of the covariance from the start, so we can assume $i \neq j$

$$\Delta S = \frac{dS}{dK_{ij}}(K_{ij} - \Sigma_{ij}) + \frac{1}{2}\frac{d^2S}{dK_{ij}^2}(K_{ij} - \Sigma_{ij})^2 + ... \tag{2}$$

The entropy of a Gaussian probability distribution has the following form:[5]

$$S = \frac{N}{2}\ln(2\pi e) + \frac{1}{2}\ln(|K|) \tag{3}$$

Starting with the first order term:

$$\frac{dS}{dK_{ij}} = \frac{\partial S}{\partial K_{ij}} + \sum_{(kl)\notin E^*,(kl)\neq(ij)} \frac{\partial S}{\partial K_{kl}}\frac{dK_{kl}}{dK_{ij}} \tag{4}$$

Where E^* is the network of covariances that are being fit, including the diagonal (meaning the variances). The elements of K that are being fit are not free parameters as they are constrained to match their experimental values, whereas the edges not in E^* are able to change, which is why we sum over them above. Let's look at the partial derivatives:

$$\frac{\partial S}{\partial K_{ij}} = \frac{1}{2|K|}\frac{\partial |K|}{\partial K_{ij}} \tag{5}$$

To obtain the derivative on the right hand side, we use the following result for the partial derivative of the determinant:[6]

$$\frac{\partial |K|}{\partial K_{ij}} = |K| \operatorname{Tr}(J \frac{\partial K}{\partial K_{ij}}) \tag{6}$$

The matrix $\frac{\partial K}{\partial K_{ij}}$ is equal to one for both the ij and ji elements, as those elements are really the same parameter due to the enforced symmetry of the covariance matrix. We can denote the mn-th element of this as:

$$\left(\frac{\partial K}{\partial K_{ij}}\right)_{mn} = \delta_{mi}\delta_{nj} + \delta_{mj}\delta_{ni} \tag{7}$$

With this, we have:

$$\operatorname{Tr}(J \frac{\partial K}{\partial K_{ij}}) = \sum_m \sum_k J_{mk}(\delta_{ki}\delta_{mj} + \delta_{kj}\delta_{mi}) = J_{ji} + J_{ij} = 2J_{ij} \tag{8}$$

Using this, we have:

$$\frac{\partial S}{\partial K_{ij}} = \frac{1}{2|K|}\frac{\partial |K|}{\partial K_{ij}} = \frac{1}{2|K|}|K| 2J_{ij} = J_{ij} \tag{9}$$

Plugging this into our total derivative expression, while noting that if $(kl) \notin E^*$, then $k \neq l$ as we fit the variances from the start:

$$\frac{dS}{dK_{ij}} = J_{ij} + \sum_{(kl)\notin E^*, (kl)\neq(ij)} J_{kl}\frac{dK_{kl}}{dK_{ij}} \tag{10}$$

Entropy maximization requires that $J_{kl} = 0$ for $(kl) \notin E^*$, so we see that the sum on the right hand side vanishes (assuming the total derivative term is well behaved), and we are left with:

$$\frac{dS}{dK_{ij}} = J_{ij} \tag{11}$$

We can now move on the the second derivative term in our expansion. We see now that

$$\frac{d^2 S}{dK_{ij}^2} = \frac{dJ_{ij}}{dK_{ij}} \tag{12}$$

We will proceed by calculating $\frac{dK_{ij}}{dJ_{ij}}$ then inverting. We can rewrite the above as follows:

$$\frac{dK_{ij}}{dJ_{ij}} = \frac{\partial K_{ij}}{\partial J_{ij}} + \sum_{(kl)\in E^*} \frac{\partial K_{ij}}{\partial J_{kl}}\frac{dJ_{kl}}{dJ_{ij}} \tag{13}$$

For the partial derivative of an inverse matrix with respect to an element of the original matrix, the following result is known for a general matrix M:[6]

$$\frac{\partial M^{-1}_{ij}}{\partial M_{kl}} = -\left(M^{-1}\frac{\partial M}{\partial M_{kl}}M^{-1}\right)_{ij} \tag{14}$$

Using this, and setting $M = J$ and $M^{-1} = K$, we have:

$$\frac{\partial K_{ij}}{\partial J_{kl}} = -\left(K\frac{\partial J}{\partial J_{kl}}K\right)_{ij} \tag{15}$$

Using similar notation as in Eqn. (7) we have, for $k \neq l$:

$$\frac{\partial K_{ij}}{\partial J_{kl}} = -\sum_m K_{im}\sum_n(\delta_{mk}\delta_{nl} + \delta_{ml}\delta_{nk})K_{nj} = -K_{ik}K_{lj} - K_{il}K_{kj} \tag{16}$$

For $k = l$ we have:

$$\frac{\partial K_{ij}}{\partial J_{kl}} = -\sum_m K_{im}\sum_n \delta_{mk}\delta_{nk}K_{nj} = -K_{ik}K_{kj} \tag{17}$$

Now we just need to find $\frac{dJ_{kl}}{dJ_{ij}}$, then we will have the full expression for the second derivative term. To make progress, we use the following trick: For an edge $(kl) \in E^*$, and $(ij) \notin E^*$ we have that K_{kl} cannot change, as it is being fit, and:

$$\frac{dK_{kl}}{dJ_{ij}} = 0 = \frac{\partial K_{kl}}{\partial J_{ij}} + \sum_{(mn)\in E^*}\frac{\partial K_{kl}}{\partial J_{mn}}\frac{dJ_{mn}}{dJ_{ij}} \tag{18}$$

We can solve the equation above to find $\frac{dJ_{mn}}{dJ_{ij}}$. We do this by treating the above as a matrix equation $A\vec{x} = \vec{b}$, where:

$$\vec{b}_{e_1} = -\frac{\partial K_{e_1}}{\partial J_{ij}}, \vec{x}_{e_2} = \frac{\partial J_{e_2}}{\partial J_{ij}}, A_{e_1e_2} = \frac{\partial K_{e_1}}{\partial J_{e_2}} \tag{19}$$

and e_1 and e_2 are edges in E^*. We can obtain all of the elements in A and b using (16). Assuming A is invertible, we have:

$$\frac{dJ_{kl}}{dJ_{ij}} = -\sum_{(mn)\in E^*} A^{-1}_{(kl),(mn)}\frac{\partial K_{mn}}{\partial J_{ij}} \tag{20}$$

We can summarize

$$\frac{dK_{ij}}{dJ_{ij}} = \frac{\partial K_{ij}}{\partial J_{ij}} - \sum_{(kl)\in E^*}\frac{\partial K_{ij}}{\partial J_{kl}}\sum_{(mn)\in E^*}A^{-1}_{(kl),(mn)}\frac{\partial K_{mn}}{\partial J_{ij}} \tag{21}$$

We now have all that we need. Eqn. 2 becomes:

$$\Delta S \approx J_{ij}(K_{ij} - \Sigma_{ij}) + \frac{1}{2}\left[\frac{\partial K_{ij}}{\partial J_{ij}} - \sum_{(kl)\in E^*}\frac{\partial K_{ij}}{\partial J_{kl}}\sum_{(mn)\in E^*} A^{-1}_{(kl),(mn)}\frac{\partial K_{mn}}{\partial J_{ij}}\right]^{-1}(K_{ij} - \Sigma_{ij})^2 \quad (22)$$

We are evaluating this for an edge (ij) that has not yet been added to the network, so $J_{ij} = 0$, meaning that the first order term in the error vanishes. With the elements of the matrix A and the partial derivatives given previously, everything in this expression now depends only on the elements of the current model covariance K (that is, the one found at the prior step without the edge (ij) constrained), and the data covariance Σ. While the final result may not look pretty, it can be numerically calculated much faster than it would take to actually find the true maxent model with the same edge added. A comparison of predicted versus actual entropy drops can be found in Fig. S1 and we find good agreement.

3 Predicting the information gained from adding a constraint

Here we compare our predictions for the true entropy drops, obtained by finding the corresponding maxent model and calculating its entropy, for each of the possible edges that could be added to the optimal network with 100 edges added for the combined fMRI data. In Fig. S1a we see the predicted and true information gains versus the error on the prediction of the corresponding element of the covariance before that edge was added. Notice the approximately quadratic shape, which is assuring given that the expansion in our prediction goes to second order in the error, with the linear term being zero. In Fig. S1b we see the predicted information gains plotted versus the true gains, finding good agreement.

4 Comparison of our method of selecting edges to heuristics

One could try to choose which correlations to add to the model as we build it using some heuristic, for example picking the largest correlations in magnitude, as we did in the main text. There are many other heuristics one could use. In figure S2 we compare the optimal, random, and max($|\Sigma_{ij}|$) methods from the main text to a list of other methods, all computed on the full data: max(Σ_{ij}), max

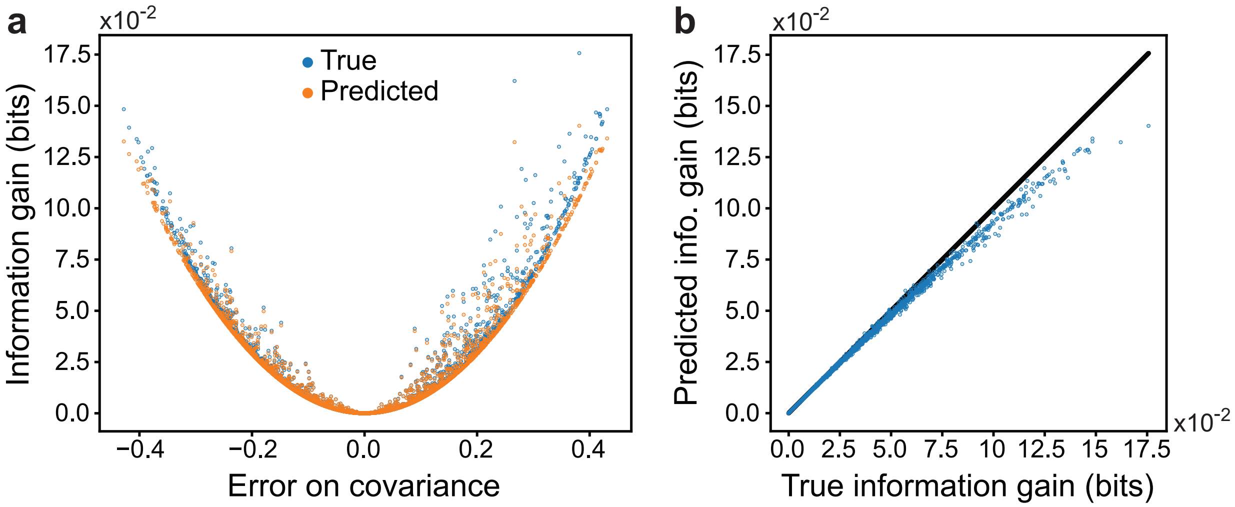

Fig. S1 | Information gained from adding a constraint can be predicted accurately. a, Predicted and true information gains from adding each possible next constraint to the optimal network with 100 correlations fit, for the combined fMRI data, versus the error on the prediction of the corresponding covariance before it is fit. For each of true and predicted, each point represents one of the 4850 possible correlations that could be added to become the 101st edge in the network. **b**, Predicted information gain from adding each possible next constraint to the optimal network with 100 correlations fit, for the combined fMRI data, versus the true information gain. Each point represents one of the 4850 possible correlations that could be constrained. The diagonal line represents a slope of one.

partial correlation in magnitude, max signed partial correlation, max($|\Sigma_{ij}^{-1}|$), max(Σ_{ij}^{-1}), min(Σ_{ij}^{-1}), and largest mutual informations, where Σ^{-1} is the data precision matrix. For Gaussians, the partial correlation is $\frac{-\Sigma_{ij}^{-1}}{\sqrt{\Sigma_{ii}^{-1}\Sigma_{jj}^{-1}}}$, which measures the correlation between regions i and j when controlling for the indirect dependencies between them through all of the other regions in the brain. We see varying degrees of success, with the largest absolute partial correlations and the max($|\Sigma_{ij}^{-1}|$) working the best.

5 Additional brain-system level structure analysis

In main text Fig. 5a, we see the brain-system level structure of optimal models compared to the random expectation for all tasks. Specifically we examine the difference in connection density between/within systems for the optimal model and random expectation for networks with approx-

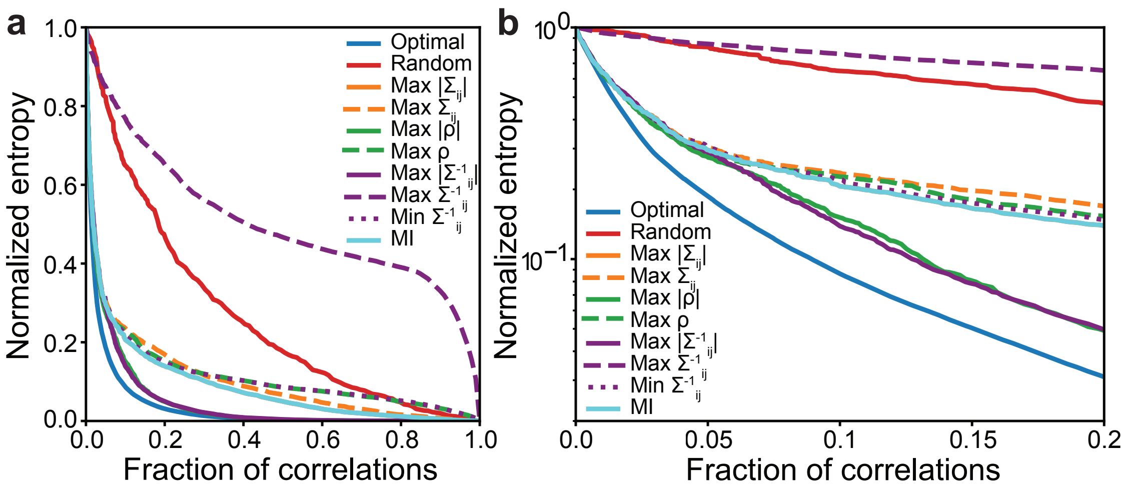

Fig. S2 | Model entropy for heuristic methods. **a**, Normalized model entropy of models of the combined fMRI data for various heuristics for selecting correlations to add to the model, compared to optimal and random methods. Here ρ stands for partial correlation, Σ is the data covariance, Σ^{-1} is the data precision matrix, and MI stands for mutual information. **b**, Same as **a** but with log scale y-axis and for fraction of correlations between 0 and 0.2

imately 10% of correlations. In Fig. S3a we look at the same but for each task including rest. One consistent feature is that there are more edges within systems than would be expected at random for all systems, which indicates that the coarse-graining of regions into systems was reasonable. One might also be interested to see which regions are more or less likely to be informative for a certain task as compared to rest. In Fig. S3b we see the difference of connection density between each task and rest, again for 10% of correlations. We see some unique differences for each task, although we will not attempt to interpret them here.

6 Model predictions relative to bootstrap errors

The high compressibilities seen in main text Fig. 3 suggest that with only a small fraction of correlations constrained, we should be able to accurately predict the rest. In order to get a sense for how accurate the predictions really are, we should measure the error relative to the inherent scale of uncertainties in the correlations themselves. To do this, we use the standard deviation of a correlation across bootstrap samples of the data (see Methods). We calculate the maximum

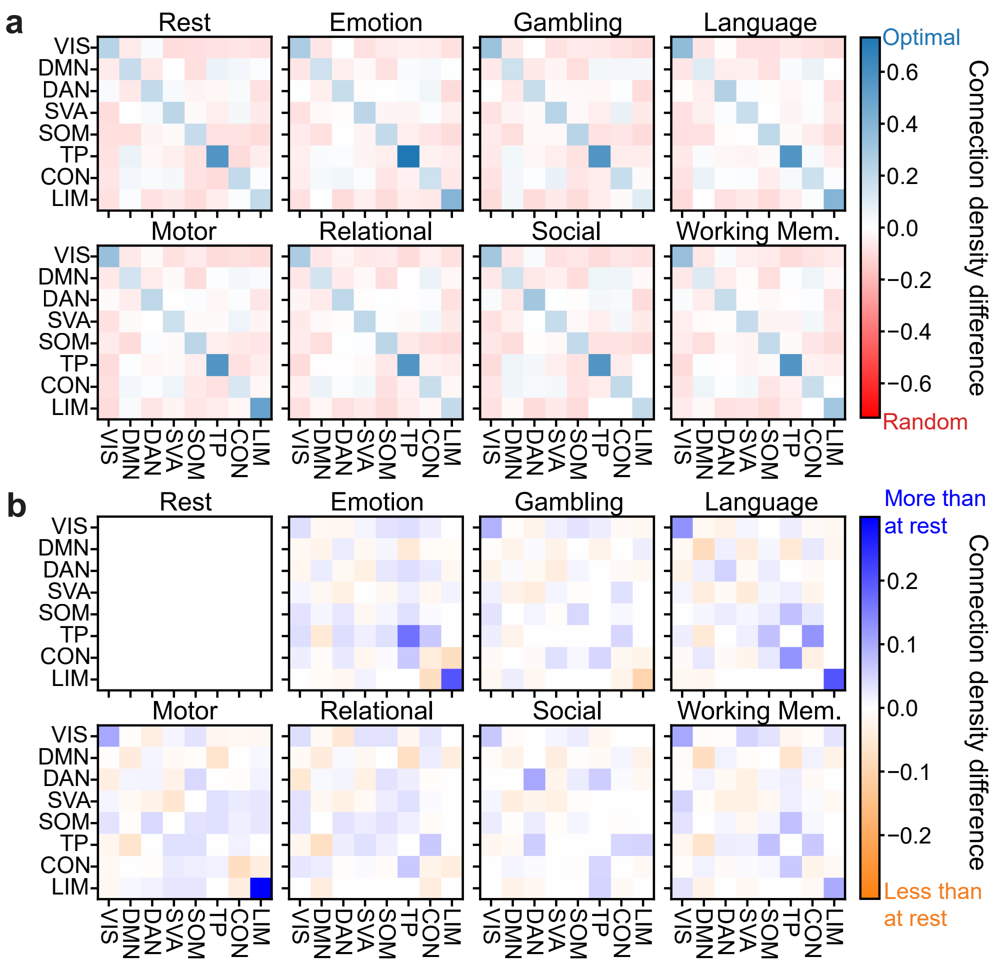

Fig. S3 | System level structure of optimal models. **a**, Difference in connection density between/within systems for the optimal model (blue) and random expectation (red) for tasks, with approximately 10% of correlations **b**, Difference of connection density between each task and rest, with approximately 10% of correlations. Blue corresponds to higher density in the task than at rest, and orange corresponds to less.

error of our models for the combined data, for tasks, and for subjects on correlations not being fit in the models, divided by the bootstrap standard deviation of the corresponding correlation, with the results shown in Fig. S 4. We see that for the combined data, we need around 85.86% of the edges to predict the remaining correlations to within two bootstrap standard deviations. This is perhaps not surprising given that each pair of regions may exhibit a significant correlation in at least one subject or task. If we focus on specific tasks, it takes a significantly smaller fraction, between 10.2% and 12.73% of the correlations added, to predict the others within errors. For

individual subjects, it takes between 47.47% and 60.61% of the correlations. In all cases, we see a large decrease in the max error over bootstrap standard deviation for a relatively small fraction of edges, meaning that the sparse networks of covariances are able to give the majority of the reduction in prediction error. We would expect that once the predictions are within errors, the model is capturing most of the available information, and we see that this is indeed the case in Fig. S 4e. In summary, we find a large decrease in normalized prediction errors for a small fraction of correlations across subjects, tasks, and combined data, with the point at which the predictions are within errors changing significantly for different ways of slicing the data.

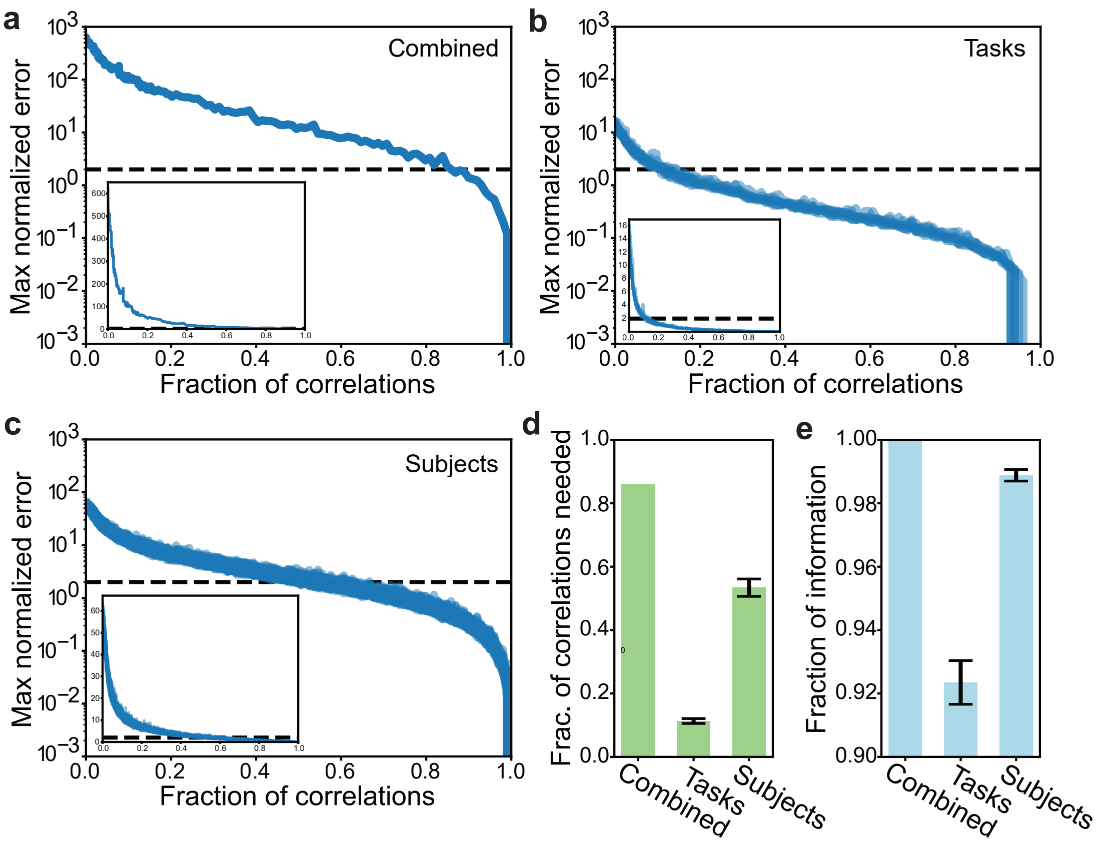

Fig. S4 | Maximum error on unconstrained covariances relative to bootstrap standard deviation. a, Maximum of errors on unconstrained covariances divided by corresponding bootstrap standard deviation for data combined across both subjects and tasks. Dashed lines in a-c represent value of 2. Inset shows linear-linear plot of same results. **b**, Same as in (a) for data combined across subjects for a given task. **c**, Same as in (a) for data combined across tasks for a given subject. **d**, Fraction of edges added at which threshold of 2 is crossed. **e**, Fraction of possible entropy drop at the time when threshold is crossed.

7 Parcellation with 200 nodes

The data used in all parts of the main text and supplement, other than this supplementary section, uses a parcellation of the brain into 100 parcels. One might be concerned that using only 100 parcels biases us to find high compressibility. We find that this is not the case, and that using more parcels actually gives a slightly higher compressibility. In Fig. S5 we compute model entropy curves and compressibilities for data combined across all subjects and tasks as before, but now using a parcellation with 200 parcels. Here the compressibility is 0.98, higher than the compressibility of 0.96 found for the parcellation with 100 nodes.

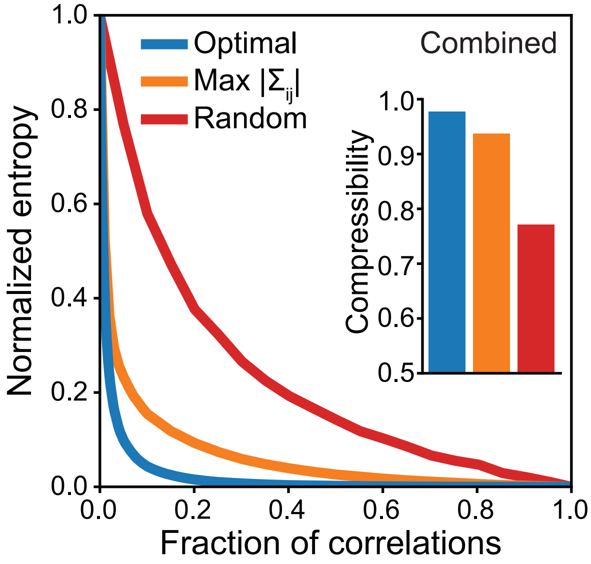

Fig. S5 | Normalized entropy $\tilde{S}_G$ for parcellation with 200 nodes, as a function of the fraction of correlations in G for the optimal networks (blue), strongest correlations $|\Sigma_{ij}|$ (orange), and random networks (red).

8 Tolerances and Bootstrapping

To find reasonable error tolerances for the maxent algorithms above, we bootstrap sample the data 100 times and calculate the model covariance and precision matrix for each of those bootstrap sample timeseries. We then find the standard deviation of the elements of each matrix. We set the error tolerance as the smallest standard deviation divided by 100. For task models we used the smallest tolerances across tasks for all of the tasks due to a reasonably large difference in tolerances

between tasks. For the subject models, we used each individual's tolerances.

Supplemental Information References

1. Nguyen, H. C., Zecchina, R. & Berg, J. Inverse statistical problems: from the inverse ising problem to data science. *Adv. Phys.* **66**, 197–261 (2017).
2. Lynn, C. W., Yu, Q., Pang, R., Palmer, S. E. & Bialek, W. Exact minimax entropy models of large-scale neuronal activity. *Phys. Rev. E* **111**, 054411 (2025).
3. Lynn, C. W., Yu, Q., Pang, R., Bialek, W. & Palmer, S. E. Exactly solvable statistical physics models for large neuronal populations. *Phys. Rev. Res.* **7**, L022039 (2025).
4. Uhler, C. Gaussian graphical models: An algebraic and geometric perspective (2017). URL `https://arxiv.org/abs/1707.04345`. `1707.04345`.
5. Cover, T. M. & Thomas, J. A. *Elements of Information Theory* (Wiley, 2006), 2nd edn.
6. Magnus, J. R. & Neudecker, H. *Matrix Differential Calculus with Applications in Statistics and Econometrics* (Wiley and Sons, 2019), 3rd edn.
